# Dual Network Hydrogel with High Mechanical Properties, Electrical Conductivity, Water Retention and Frost Resistance, Suitable for Wearable Strain Sensors

**DOI:** 10.3390/gels9030224

**Published:** 2023-03-14

**Authors:** Chen Miao, Penghui Li, Jiangdong Yu, Xuewen Xu, Fang Zhang, Guolin Tong

**Affiliations:** 1 Jiangsu Co-Innovation Center of Efficient Processing and Utilization of Forest Resources, Jiangsu Provincial Key Laboratory of Pulp and Papermaking Science and Technology, Nanjing Forestry University, Nanjing 210037, China; 2 College of Food Science and Light Industry, Nanjing Tech University, Nanjing 211800, China

**Keywords:** conductive hydrogel, TEMPO oxidized nanocellulose, water resistant, frost resistance, sensor

## Abstract

With the progress of science and technology, intelligent wearable devices have become more and more popular in our daily life. Hydrogels are widely used in flexible sensors due to their good tensile and electrical conductivity. However, traditional water-based hydrogels are limited by shortcomings of water retention and frost resistance if they are used as the application materials of flexible sensors. In this study, the composite hydrogels formed by polyacrylamide (PAM) and TEMPO-Oxidized Cellulose Nanofibers (TOCNs) are immersed in LiCl/CaCl_2_/GI solvent to form double network (DN) hydrogel with better mechanical properties. The method of solvent replacement give the hydrogel good water retention and frost resistance, and the weight retention rate of the hydrogel was 80.5% after 15 days. The organic hydrogels still have good electrical and mechanical properties after 10 months, and can work normally at −20 °C, and has excellent transparency. The organic hydrogel show satisfactory sensitivity to tensile deformation, which has great potential in the field of strain sensors.

## 1. Introduction

With the progress of science and technology, intelligent wearable devices are becoming more and more popular in our lives [1,2,3,4]. However, the flexibility and sensitivity of traditional sensors are insufficient, and the comfort level is poor in the wearing process. Therefore, it is very important to develop flexible sensors [5]. Flexible sensors have attracted more and more attention due to their inherent transparency [6,7], adjustable mechanical properties [8,9] and wearable properties [10,11,12]. But, if traditional water-based hydrogels are used as the application materials of flexible sensors, there will be many limitations [13]. On the one hand, traditional water-based hydrogels contain a large amount of water, which will inevitably freeze at sub-zero temperature, becoming rigid, fragile and non-conductive, thus inhibiting the application of water-based hydrogels at low temperatures. On the other hand, even indoors, hydrogels inevitably dry out as water evaporate [14,15,16]. The loss of flexibility, tensile property and electrical conductivity caused by freezing and drying are two inherent problems of water-based hydrogels, which seriously damage their stability, durability and application range of preparation devices. Therefore, it is of great significance to solve the freezing resistance [17] and water retention [18] properties of hydrogels.

An effective method to improve the freezing resistance of hydrogel is to permeate organic solvents [19]. Inspired by the oil-water system, Rong et al. [20] reported an antifreezing conductive organic hydrogel using H_2_O/Ethylene (Eg) dual solvent as the dispersion medium. The addition of Eg is conducive to the form of hydrogen bonds with H_2_O, which prevents the formation of ice crystals. The hydrogel have steady mechanical properties and electrical conductivity in the temperature range of −55~44.6 °C. Good elasticity and excellent electrical can conduct electricity below −40 °C. Li et al. [21] reported a H_2_O/DMSO system in which hydrogel can operate normally at −50~50 °C. The addition of salt solution can also enhance the frost resistance of hydrogels. Chen et al. [22] mixed Glycerol (Gl) with NaCl and carried out solvent replacement on hydrogels, which not only further improved the freeze-resistance and electrical conductivity of hydrogels, but also gave them good water retention properties.

Double network (DN) [23,24] hydrogels have attracted much focus because of their high mechanical strength and flexibility, with tensile stress up to 10 MPa and strain up to 2000% [25]. As a promising material, polyacrylamide (PAM) has been widely synthesized into hydrogels to obtain hydrogel with high toughness [26]. Cellulose is not only renewable in nature, but also a biocompatible polymer [27]. As a composite enhancement phase, TEMPO-Oxidized Cellulose Nanofibers (TOCNs) have got extensive focus because of their big aspect ratio and tangles. TOCNs are cellulose modified derivatives resulting from the oxidation of hydroxymethyl at the C_6_ site of cellulose to carboxyl groups [28]. TOCNs can be used as a green filler to enhance polymer properties [29]. Hu et al. [13] found that TOCNs can enhance the mechanical properties of polymer composites and ionic conductivity. Since the -OH of C_6_ group of cellulose fibers can be translated to -COOH of C_6_ groups, the negative surface of TOCNs is benefit to counter-ion mobility, thus enhancing ionic conductivity. TOCNs show double effects where they enhance mechanical properties and ionic conductivity, providing a way for addressing the important weigh and balance between mechanical properties and electrical conductivity of ionic conductive antifreeze hydrogels [30].

**Figure 1:**
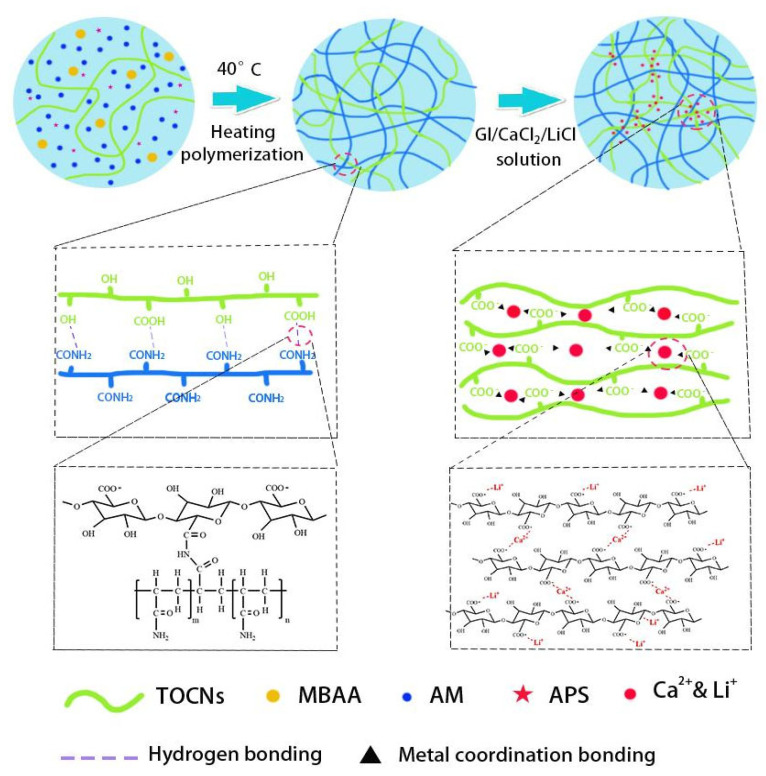
PAM/TOCNs/LiCl/CaCl_2_/Gl hydrogel mechanism diagram.

In this study, PAM/TOCNs organic hydrogels were synthesized by free radical polymerization and then immersed in 1 M LiCl, 1 M CaCl_2_, 1:1 deionized water (DI) and Gl, and its theory as shown in Figure 1. In order to obtain excellent mechanical properties and ionic conductivity of the hydrogel, PAM/TOCNs hydrogel was immersed in a solution containing LiCl and CaCl_2_ at a ratio of 1:1 water and Gl for 2 h. In order to construct hydrogels with high tensile and toughness, acrylamide (AM) was selected as monomer, N,N′-methylene diacrylamide (MBAA) as crosslinking agent, and ammonium persulfate (APS) as initiator. The -COOH group of TOCNs forms hydrogen bonds with the amide group of AM, and metallic bonds are formed between the carboxyl group on TOCNs and Ca^2+^ and Li^+^ in LiCl/CaCl_2_/Gl solvent. The hydrogels with high conductivity, high freeze resistance and high moisture retention, and excellent transparency, organic hydrogels show content sensitivity to tensile stress, and can be fitted with a perfect strain sensor to monitor people movement. 

## 2. Results and Discussion

### 2.1. Characterization

**Figure 2:**
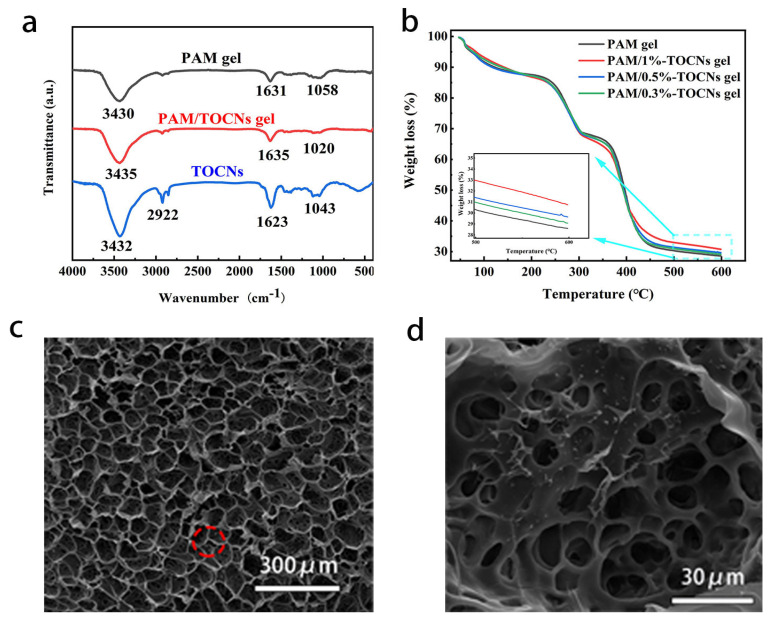
(**a**) FTIR spectra of PAM/TOCNs hydrogel (red line) and PAM/TOCNs (black line). (**b**) TGA curves of PAM,PAM/0.3%-TOCNs, PAM/0.5%-TOCNs and PAM/1%-TOCNs hydrogel. (**c**) SEM images of PAM/1%-TOCNs hydrogel after freeze-drying. (**d**) Zoomed in SEM image of the red spotty of the PAM/1%-TOCNs hydrogel.

The structures of TOCNs, PAM and TOCNs/PAM hydrogels were characterized by FT-IR [31] (Figure 2a). PAM hydrogel has an obvious wide absorption peak near 3430 cm^−1^ [32], which belongs to the N-H stretching vibration of PAM. The corresponding C=O stretching vibration of PAM is detected near 1631 cm^−1^ [33], and another main absorption peak is detected near 1058 cm^−1^. This is caused by the in-plane oscillation of NH_2_ [34,35]. The main absorption peak of TOCNs is about 3432 cm^−1^, which is due to the tensile vibration caused by O-H on the cellulose molecular chain [36,37]. The absorption peak at 2900 cm^−1^ is due to the tensile vibration of C-H, and the absorption peak at 1623 cm^−1^ is due to the tensile vibration of C=O [38]. Compared with pure PAM hydrogel, the absorption peak positions of N-H, C=O and NH_2_ of TOCNs/PAM composite hydrogels were 3430 cm^−1^, 1635 cm^−1^ and 1020 cm^−1^, and the peak positions did not change significantly. This may be because the characteristic absorption peak of TOCNs overlaps with the PAM related absorption peak. In addition, there is no new vibration absorption peak in the TOCNs/PAM spectrum, indicating that there is only physical interaction between PAM and TOCNs, and no new chemical bond is formed. NH_2_ absorption vibration peak shifted from 1058 cm^−1^ to 1020 cm^−1^, C=O stretching vibration peak moved from 1631 cm^−1^ to 1635 cm^−1^, and N-H stretching vibration absorption peak moved from 3430 cm^−1^ to 3435 cm^−1^. This may be on account of the form of hydrogen bonds between PAM and TOCNs, leading to the displacement of peak positions.

PAM/TOCNs hydrogels with various TOCNs contents were characterized by thermogravimetric (TG) analysis (Figure 2b), the heat stability and remnant weight of the sample monotonically increase with the increase of TOCNs content. The strong interaction between TOCNs and PAM is shown again. 

By shooting SEM images of freeze-dried gels, we can observe that freeze-dried hydrogel has many large pore sizes (Figure 2c), among which there are many small pore sizes (Figure 2d). These connected pore sizes not only allow ions to move freely, but also play a good supporting role.

### 2.2. Mechanical Performance Test 

**Figure 3:**
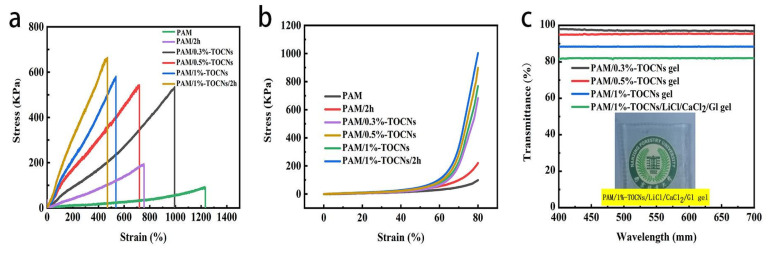
(**a**) Stress-strain curves. (**b**) The compressive stress-strain curves. (**c**) Transparency curve and image of PAM/1%-TOCNs/LiCl/CaCl_2_/Gl hydrogel film.

PAM/TOCNs hydrogels exhibit perfect mechanical properties because of the double network structure, synergistic dynamic cross-linked physical interactions, and the enhancement of TOCNs in the polymer matrix (Figure 3a). Pure PAM hydrogel exhibits a maximum tensile stress of 88 KPa and a fracture strain of 1230%. With the increase of TOCNs concentration, when the content of TOCNs is 0.3%, 0.5% and 1%, the corresponding tensile strength respectively is 524 KPa, 540 KPa and 580 KPa, and the fracture strain respectively is 1000%, 720% and 540%.The tensile strength increases with the increase of TOCNs content. The fracture strain decreases with the increase of TOCNs content, which further indicates that the hydrogen bond between TOCNs and PAM is formed. After soaking in CaCl_2_/LiCl/Gl for 2 h, the tensile strength of PAM and PAM/TOCNs hydrogels was 190 KPa and 660 KPa, and the tensile strain was 750% and 470%, respectively. After LiCl/CaCl_2_/Gl solvent immersion, PAM/TOCNs hydrogels formed chemical bonds.

PAM/TOCNs/LiCl/CaCl_2_/Gl hydrogels have excellent tensile and compressive properties. As shown in the Figure 3b, every samples have good compressive stress. When the strain is 80%, the compressive stress of PAM/TOCNs/LiCl/CaCl_2_/Gl hydrogels increases with the increase of TOCNs content, which is because of the synergistic effect of dynamic cross-linking physical interaction and the enhancement of TOCNs.

The transmittance (Figure 3c) of PAM/1%-TOCNs/LiCl/CaCl_2_/Gl hydrogel is slightly lower than PAM/1%-TOCNs hydrogel, and the transmittance of hydrogel decreases with the increase of cellulose content, all the transmittance of all hydrogels is more than 80%, the excellent transparency of the hydrogels is advantageous for wearable applications that require visualization [39,40].

### 2.3. Water Retention Capacity

**Figure 4:**
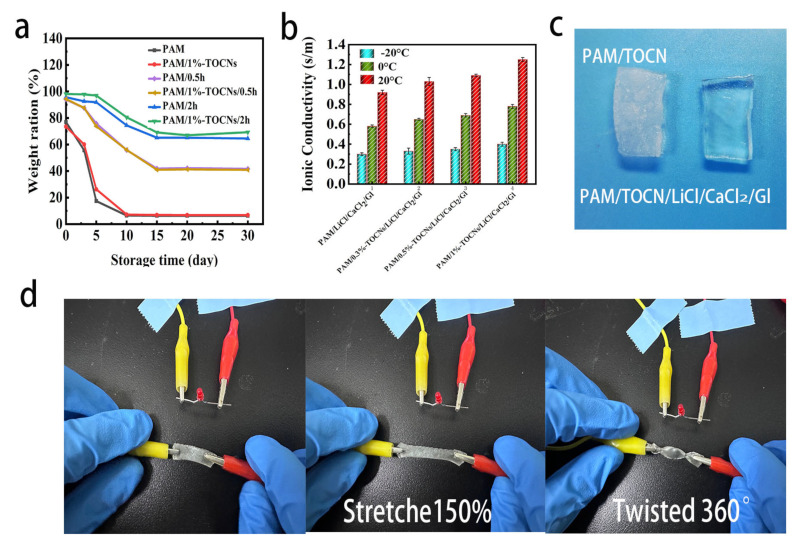
(**a**) The weight retention rate of hydrogels. (**b**) Conductivity of hydrogels variation with temperature for different TOCNs contents. (**c**) Surface morphologies of different hydrogels after 24 h at −20 °C. (**d**) Stretching and twisting of the hydrogel after 10 months.

As a special kind of intellective hydrogels, ionic conductive hydrogels have steady performance at room temperature, but the traditional ionic conductive hydrogels will lose their performance (e.g., conductivity, toughness) in harsh environment, which limits their application [41]. Wu et al. [42] developed a solvent Eg/Gl for the production of anti-freeze and anti-drying organic hydrogels with excellent stability and repeatability in the range of −18 °C–25 °C, maintaining strain sensing capability after nine months of exposure to ambient air. We soaked PAM/TOCNs organic hydrogel in a mixture of Gl/H_2_O (1:1), weighed and measured at regular intervals in a constant temperature and humidity (25 °C, 50% relative humidity) environment, recorded and calculated these hydrogels’ weight retention rate, so as to observe the water retention rate of the hydrogel (Figure 4a). The protection rates of PAM and PAM/TOCNs hydrogels on day 10 were only 6.5% and 7.2%. The slightly higher weight retention rates of PAM/TOCNs hydrogels were due to the presence of carboxyl groups in TOCNs, which formed hydrogen bonds with amino groups. After soaking in LiCl/CaCl_2_/Gl solution for 0.5 h, the weight retention rate of PAM/TOCNs hydrogel increased significantly, and stabilized at 80.5% on the 15th day after soaking for 2 h. The weight retention rate of PAM/TOCNs organic hydrogels after 2 h immersion was significantly improved due to the hygroscopic properties of Gl, which easily forms hydrogen bonds with water molecules, thus preventing the evaporation of water.

Ionic conductivity of organic hydrogels is very important for flexible electronic equipment. The change of shape of ionic conductive hydrogels under external stimuli (pressure, temperature, humidity) may cause the change of ion transport channels [43]. To improve the conductivity of the hydrogel, PAM/TOCNs hydrogel was soaked in LiCl/CaCl_2_/Gl solution to allow ions to diffuse into the hydrogel network. Wang et al. [26]. studied the optimal soaking time of hydrogels in LiCl/CaCl_2_/GI solvent. After soaking for 2 h, the cracking strength of hydrogels reached the maximum value, and the ionic conductivity and volume fraction were in a good balance. We also chose the soaking treatment time of 2 h to balance the mechanical and electrical properties of the hydrogel. We used electrochemical impedance (EIS) to characterize the ionic Conductivity of hydrogels variation with temperature for different TOCNs content. As shown in the Figure 4b, ionic conductivity of organic hydrogels increases monotonically with different TOCNs contents, and the conductivity of the organic hydrogel decreases with the decrease of temperature. The ionic conductivity of PAM/LiCl/CaCl_2_/Gl hydrogel was 0.92 S/m when the temperature is 20 °C, and the ionic conductivity of PAM/1%-TOCNs/LiCl/CaCl_2_/Gl hydrogels was 1.25 S/m, 36% higher than that of PAM/LiCl/CaCl_2_/Gl hydrogels, indicating that TOCNs enhance the ionic conductivity of hydrogels. This is because the carboxyl group on the surface of TOCNs attracts counter-ions and promotes their migration [44]. The ionic conductivity of PAM/0.3%-TOCNs/LiCl/CaCl_2_/Gl hydrogels was 0.33 S/m at −20 °C, 0.65 S/m at 0 °C, and 1.03 S/m at 20 °C. It is because at low temperatures, the movement of ions becomes slow and limited.

Compared with PAM/TOCNs organic hydrogels and PAM/TOCNs/LiCl/CaCl_2_/Gl organic hydrogels, PAM/TOCNs/LiCl/CaCl_2_/Gl organic hydrogel still showed good freezing-resistance after PAM/TOCNs hydrogels were placed in the refrigerator at a low temperature (−20 °C) for 24 h (Figure 4c). Greatly increased its tolerance to harsh conditions. PAM/TOCNs organic hydrogels changed from transparent to opaque and the presence of ice crystals was observed, while PAM/TOCNs/LiCl/CaCl_2_/Gl organic hydrogels maintained toughness and transparency though being placed at −20 °C for 24 h. That’s because a mixed solution of H_2_O and Gl reduces the freezing point of water, and the combined action of metal cations such as Li^+^ and Ca^2+^, and anions such as Cl^−^ increase the pressure in the solution and require more release. More energy can be solidified and the freezing point lowered further. Therefore, PAM/TOCNs/LiCl/CaCl_2_/Gl organic hydrogels have excellent frost resistance. This ionic conductive hydrogel with water retention and frost resistance has a broad application prospect in extreme conditions in the future [45].

After PAM/TOCNs hydrogels soaking in LiCl/CaCl_2_/Gl solution 2 h, become PAM/TOCNs/LiCl/CaCl_2_/Gl hydrogels, PAM/TOCNs/LiCl/CaCl_2_/Gl hydrogels exposed to air (25 °C, 50% relative humidity) for 10 months still maintains its sensing properties and electrical conductivity, with excellent water retention performance (Figure 4d). PAM/TOCNs/LiCl/CaCl_2_/Gl hydrogel was exposed to air for 10 months, stretched 150% and twisted 360° without structural damage. Wu et al. [42] developed a Eg/GL soaked hydrogel sensor, there was no significant decrease in sensitivity after 9 months and no structural damage after 400% strain and twisted 360° when exposed to ambient air (25 °C, 70% relative humidity (RH)) The sensitivity and mechanical properties of PAM/TOCNs/LiCl/CaCl_2_/Gl hydrogels have good stability and can be used for long-term monitoring of various human movements, it has good application prospect.

### 2.4. Adhesion Property

**Figure 5:**
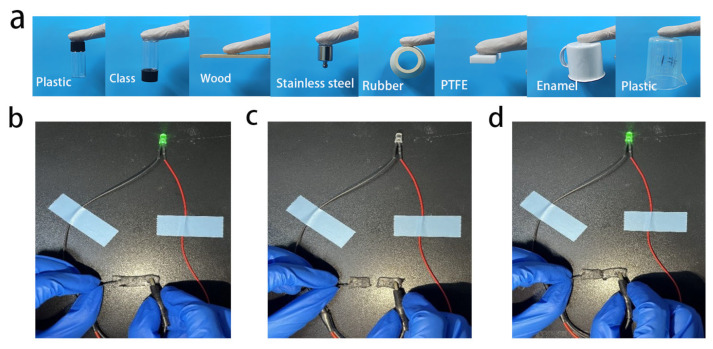
(**a**) Adhesion of hydrogels to surfaces of different materials. (**b**) Primitive hydrogel. (**c**) Cut hydrogel. (**d**) Hydrogel after bonding.

Good adhesion is also needed for wearable sensor applications. In order to evaluate the adhesion properties of organic hydrogels, the hydrogels were adhered to the surfaces of plastics, glass, wood blocks, stainless steel, rubber, PTFE and enamel, as shown in (Figure 5a). Organic hydrogels showed good adhesion to different materials. This is because PAM/TOCNs/LiCl/CaCl_2_/Gl organic hydrogels are rich in polar functional groups, such as -C=O, -OH, -COOH and -NH_2_. This allows PAM/TOCNs/LiCl/CaCl_2_/Gl organic hydrogels to interact with a variety of materials, such as hydrogen bonding, metal coordination and electrostatic effects [46].

As shown in the Figure 5b, the organic hydrogel PAM/TOCNs/LiCl/CaCl_2_/Gl can light the green LED. When the LED is cut in half, the LED is extinguished (Figure 5c), and when it is put together, the LED lights up again (Figure 5d), and the brightness of the light emitted is roughly the same as that when the hydrogel is not cut. It indicates that it has good self-repair ability [47]. It is mainly from the reversibility of the hydrogen bond between the PAM and TOCNs chains when the two severed hydrogels come into contact. The PAM and TOCNs chains spread from both sides to the interface, re-forming hydrogen bonds. In addition, according to the electrolyte solution theory, ions combine with water molecules, forcing PAM and TOCNs chain groups at the interface to contact each other, resulting in a more stable hydrogen bond [48].

### 2.5. Sensing Performances and the Application for Monitoring Human Motions

**Figure 6:**
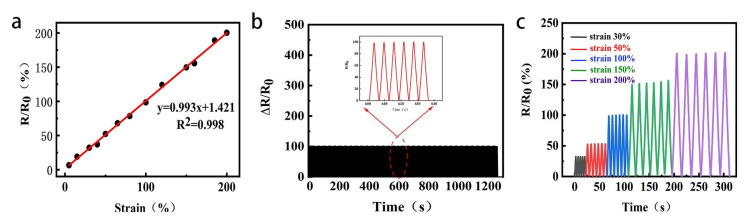
(**a**) Relative resistance change linear fitting curve of the hydrogel with varying tensile strain. (**b**) The curve of relative resistance changes of the strain sensor for 220 times under cyclic stretching-releasing testing up to 100% strain. (**c**) The curve of relative resistance change of the hydrogel with varying tensile strain.

As shown in the Figure 6a, is the change of the resistance of the hydrogel under different tensile strains, and its gauge factor (GF) is 0.99, showing a high sensitivity, and no obvious change in the resistance was seen, indicating its potential application in strain sensors.

Stability and durability are the two key factors for the long-term operation of the wearable strain sensor. The cyclic durability test is shown in the Figure 6b. PAM/TOCNs/LiCl/CaCl_2_/Gl hydrogel is tested in the 0–100%–0% cycle experiment, the number of cycles is more than 220 times, and the response of the strain sensor is nearly unchanged. Wang et al. [26] prepared a alginate/PAAm dual network hydrogels, it holds and has no big degradation after 500 tensile cycles under 50% strain. Those results show that the strain sensor can work normally after multiple cycles of loading, It has broad prospect for future application.

PAM/TOCNs/LiCl/CaCl_2_/Gl hydrogel exhibit stable sensing properties during continuous deformation, and the relative resistance change rate increases from 33 to 200 with the tensile ratio increasing by 30% from 200% (Figure 6c). In each cycle, the resistance is almost constant after the tensile strain is released.

**Figure 7:**
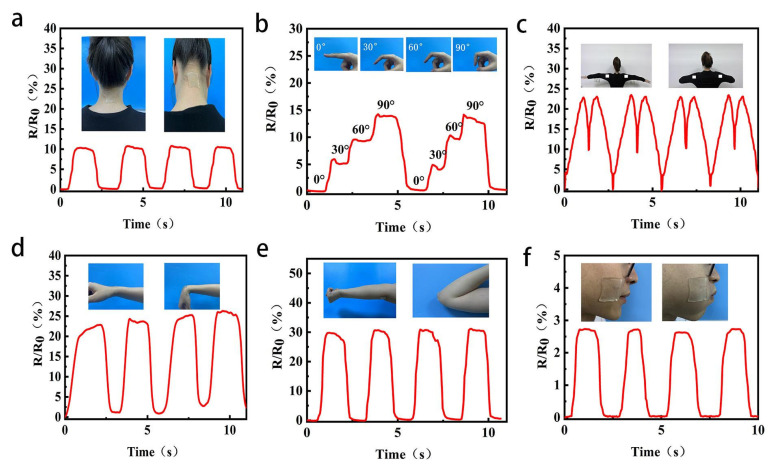
Different movement activities of PAM/TOCNs/CaCl_2_/LiCl/Gl hydrogels as wearable sensors’ photographs. (**a**) neck; (**b**) finger; (**c**) shoulder; (**d**) wrist; (**e**) elbow; (**f**) cheek.

To demonstrate this, we attach the strain sensor of PAM/TOCNs/LiCl/CaCl_2_/Gl hydrogel directly to the joint or muscle to monitor human movement. These include neck flexion (Figure 7a), finger flexion (Figure 7b), shoulder movement (Figure 7c), wrist flexion (Figure 7d), elbow flexion (Figure 7e), occlusal movement (Figure 7f). These figures show two motion states of human body. It can be seen that the strain sensor of PAM/TOCNs/LiCl/CaCl_2_/Gl hydrogel can respond to motion quickly and repeatedly, and the response of different movements is different. For example, the change in relative resistance is 15 for a bent finger and 30 for a bent elbow. This is due to the elongation of the hydrogel, which elongates the ion channels, resulting in an increase in the relative resistance change rate of the hydrogel. In addition, for the same motion, the fluctuation amplitude of each motion curve may not be exactly the same. This is because the amplitude of each motion is slightly different, which further illustrates the high sensitivity of the strain sensor of PAM/TOCNs/LiCl/CaCl_2_/Gl hydrogels. Based on the human motion monitoring demonstration above, hydrogel sensors show great potential and are used as wearable devices for high sensitivity and high reliability monitoring of human activities.

## 3. Conclusions

In summary, PAM/TOCNs/LiCl/CaCl_2_/Gl organic hydrogels with high strain sensing properties, mechanical strength, conductivity, frost resistance and water retention were prepared by a simple solvent substitution strategy in this paper, which showed excellent tensile property (tensile strength of 660 kpa, fracture strain up to 1000%). High sensitivity (GF 0.99 at 200% tensile rate), excellent water retention (weight retention rate stable at 80.5% after 15 days), good frost resistance temperature (−20 °C), high light transmittance (all up to 80%), electrical conductivity (ionic conductivity 1.25 S/m), Conductive cycle stability (up to 220 cycle tensile tests on 100% cycles), good adhesion (adhesion on plastic, glass, wood blocks, stainless steel, rubber, teflon and enamel surfaces). PAM/TOCNs/LiCl/CaCl_2_/Gl organic hydrogels can be used as flexible wearable strain sensors, which have great potential in electronic skin, wearable devices and smart clothing.

## 4. Materials and Methods

### 4.1. Materials

All chemicals obtained from commercial suppliers were not further purified. Acrylamide (AM), N,N′-methylenebisacrylamide (BMA), ammonium persulfate (APS), Glycerol (Gl), analytical grade calcium chloride (CaCl_2_), lithium chloride (LiCl), deionized water (DI), Tempo Oxidized Cellulose (TOCNs), were purchased from Tianjin Wood Sprite Technology Co., Ltd. (Tianjin, China).

### 4.2. Sample Preparation

Add a certain amount of deionized water (DI) to 1 g TOCNs and dissolve it in a 55 °C water bath, and ultrasonic for 30 min until transparent in ultrasonic crushing. Add 10% AM, 0.01%BIS, and 0.3%APS to DI and dissolve it, stir magnetically for 20 min, mix the two solutions, and fill the mixture to 50 g with DI. After stirring for 5 min, the polymerization reaction was terminated by heating to 40 °C under the protection of nitrogen for 40 min. Finally, the obtained double-network hydrogels were immersed in 1 M LiCl, 1 M CaCl_2_, 1:1 DI and Gl for different times, the sample thickness is 1 mm.

### 4.3. Characterization

#### 4.3.1. Fourier Infrared Spectroscopy, UV-Visible Spectroscopy

The Fourier Transform Infrared spectrometer (FTIR) used a NICOLET 6700 spectrophotometer (Brucker GMBH, Denkendorf, Germany) for the sample range of 500 to 4000 cm^−1^. The spectrum obtained an average of 64 scans at a resolution of 4 cm^−1^. The transmittance of organic hydrogels was characterized by an ultraviolet-visible photometer (Agilent Technologies Inc., Santa Clara, CA, USA) at a scanning rate of 100 nm/min from 400 nm to 700 nm, and the sample thickness was 1 mm.

#### 4.3.2. Field Emission Scanning Electron Microscopy

Field emission scanning electron microscopy (FE-SEM, JSM-7600F, Japan Electronics Co., Ltd., Tokyo, Japan) was used to observe the morphology of the samples.

#### 4.3.3. Thermal Stability Test

The hydrogel sample was freeze-dried into a dry gel for later use. GLA209F1 TG analyzer (Shanghai, China) was used to detect and record the samples at different temperatures. The test heating conditions were as follows: in N_2_ environment, the temperature range was 25 °C to 600 °C, and the heating rate was 10 °C/min.

#### 4.3.4. Mechanical Performance Test

The hydrogel samples of PAM/TOCNs/CaCl_2_/LiCl/Gl were prepared in dumbbell shape with a length of 75 mm, a width of 25 mm and a thickness of 2 mm, A_0_ is the base area. The tensile test was carried out on the Shimadzu universal tensile machine (AGS-X) (Columbia, MD, USA) at the speed of 20 mm/min until the hydrogel was pulled off to obtain the compressive stress and tensile strength, P is the load and it’s calculated according to the following formula:

$$\sigma =\frac{P}{A_{0}}$$

elongation at fracture of the hydrogel sample, and it’s calculated according to the following formula:

$$\delta =\frac{\Delta L}{L_{0}}$$

To ensure the accuracy of the experiment, five groups of experiments were conducted for each sample and the average value was taken.

#### 4.3.5. Determination of Frost Resistance

PAM/TOCNs/CaCl_2_/LiCl/Gl hydrogel was placed in the refrigerator (−20 °C, 0 °C) for 24 h, and was quickly removed to measure the conductivity with CHI660E electrochemical workstation (Chenhua, Shanghai, China).

#### 4.3.6. Determination of Water Retention

The water retention performance of the hydrogel was expressed by the weight retention rate (W_r_). The hydrogel was made into a cylindrical shape of 20 mm in length and 15 mm in diameter, soaked in the glycerin and water mixture solution (1:1) for 2 h, dried the surface solution, and weighed the original weight, recorded as W_0_ (g). They were placed in an environment with a temperature of 25 °C and a humidity of 50, and weighed at a distance of 1 Day, 2 Days, 5 Days, 10 Days, 15 Days, 20 Days and 30 Days respectively, and denoted as W_t_ (g). For the two samples, three independent samples were taken to calculate the average value, and the formula:

$$W_{r}=\frac{W_{r}}{W_{0}}\times 100\%$$

#### 4.3.7. Electrochemical Test

The electrochemical impedance spectra (EIS) of all organic hydrogels were measured at CHI660E Electrochemical workstation (Chenhua, Shanghai, China), and hydrogels with a height of 70 mm and a diameter of 115 mm. The organic hydrogel was sandwiched between two pieces of copper foil for measuring the resistance of the hydrogel. The X-intercept could be used as the impedance of hydrogels. The ionic conductivity is calculated by L/RA, where the L is the thickness of the hydrogel, R is the impedance value, and A is the contact area of the hydrogel.

#### 4.3.8. Sensing Performance Test of Hydrogel Sensor

The sensor’s monitoring of human movement is done with the help of a volunteer (neck flexion, finger flexion, shoulder movement, wrist flexion, elbow flexion, and occlusal movement). Through a digital multimeter (Keithley Instruments DMM6500, Solon, OH, USA) records the resistance of the hydrogel under different strains. The relative change of resistance during different movements was calculated by ΔR/R × 100%. Where ΔR and R are the resistance variation and resistance under the original strain, respectively, ε is the applied strain. The sensitivity gauge factor (GF), it’s calculated according to the following formula:

$$\mathrm{GF}=\frac{\Delta R/R_{0}}{\varepsilon }\times 100\%$$

## Data Availability

The data presented in this study are available in the article.
